# Pandemic and protest in 2020: Questions and considerations for social work research

**DOI:** 10.1177/1473325020973315

**Published:** 2021-03

**Authors:** Kimberly D Hudson, Gita R Mehrotra

**Affiliations:** Fordham University Graduate School of Social Service, New York, NY, USA; Portland State University School of Social Work, Portland, OR, USA

**Keywords:** Critical reflection, knowledge, methodology, reflexivity, scholarship, social justice

## Abstract

The convergence of the COVID-19 pandemic and social/political protest concerning structural anti-Black racism marks a moment for deep reflection and revision of many taken-for-granted assumptions about our research and academic lives as social work scholars. In this reflexive essay we, as two non-Black qualitative social work scholars, explore some of the questions and considerations for social work research that have surfaced since the emergence of these complex social, political, and economic crises. We organize our reflection around *what* we study, *why,* and *how* we go about studying it. We then offer a discussion of various constraints and challenges that emerge in this type of reflective scholarly practice, including an analysis of how contexts of white supremacy culture and neoliberalism shape social work scholarship. We close the essay with a number of recommendations for further reflection for social work scholars, such as reviewing research practices, seeking external research funding, practicing reflexivity, interrogating assumptions about knowledge production, self and community care, and integrating scholarly work into social work curriculum.

When we first saw the call for papers for this special issue in Spring 2020, we were in the midst of the COVID-19 pandemic and its immediate impacts. At that time, it felt like we were in a collective trauma unlike anything many of us had previously experienced. However, so much has happened in the weeks and months since then. At present, we are deeply experiencing multiple complex crises: the COVID-19 pandemic, the economic impacts of mass unemployment, and the crisis of structural and systemic racism. As the context of the pandemic continues, we are also in the midst of massive protests and uprisings in response to police violence against Black people and structural anti-Black racism. We are seeing thousands of people around the world rising up and risking exposure to COVID-19 and further violence from law enforcement to be part of these political protests. As has been said by many activists, this is a *movement*, not only a moment.

There is no question that we are in a critical socio-political and cultural moment/movement that holds in it both great tragedy and tremendous possibility. The crisis of the coronavirus pandemic over these past months has made visible the deep social inequities of our times—we have seen the disproportionate ways the virus directly and indirectly impacts Black, brown, poor, aging, and disabled bodies. We have also seen, in the murder of George Floyd, Breonna Taylor, and many others, the ways that police brutality and other forms of state-sanctioned violence continuously target Black bodies. The historical time we are in now demonstrates the many ways that the coronavirus pandemic and the pandemic of structural racism are interconnected and constitute two simultaneous public health crises.

While we are at a unique historical juncture, it is also critical to remember that these conversations and issues are not new. We know that systemic oppression, police brutality, health disparities, and economic inequity are deep-rooted in our society and always have been. We also know that there have consistently been movements of resistance, activism, resilience, and community care working to address these issues over centuries. Many of these efforts have been led by communities themselves and by people who are most affected by these injustices. Within social work, we also have numerous examples of scholars from around the world who have contributed in important ways to resistance movements and to developing, implementing, and studying interventions into persistent social, cultural, and structural inequities. In addition, social work has played an important role in addressing global health, health inequities, and social determinants of health, which are all critical to the current COVID-19 moment. Despite the fact that little we are seeing right now is new, many people, particularly non-Black people and people with varying degrees of privilege, are feeling the truths about structural oppression, health inequities, and injustice in new ways, and perhaps with a different sense of urgency since the pandemic started.

From our perspective, there is no question that this historical moment should give pause to all of us, and to social work as a field. For us, as qualitative scholars and educators who are deeply committed to anti-racist work and building a field with liberatory potential, this moment/movement is, frankly, overwhelming. Specifically, as two non-Black scholars/academics who live and work at the intersections of multiple identities and experiences and who are committed to racial, social, gender, and economic justice, this time has also raised important questions about the role and possibilities of social work and social work scholarship. It is a critical time for us to be continuously self-reflexive and ask ourselves hard questions as scholars, educators, activists, and community members. We do not have easy answers; however, we are invested in engaging the questions ourselves and with the larger field as part of our process. The questions that this moment/movement raises for us are profound, emergent, and multidimensional. How do we make meaning of this time as qualitative social work scholars who are personally and professionally committed to racial, economic, and social justice? How do we use our time, resources, knowledge, and skills to further social change and a truly justice-centered social work? What do these crises of COVID-19 and structural racism mean for how we think about our research and scholarship, particularly within the constraints of the institutional structures that we work within? In this essay, we raise questions about what we study and how, the relationship between our roles as scholars and broader systemic issues, and potential tensions in navigating knowledge production within social work and the larger the academic industry. While we are both qualitative researchers and speak from our own experiences, the issues and questions raised in this essay have relevance to all social work scholars regardless of methodological or epistemological orientation.

## What do we study now, and why?

Reflecting on the pandemic context in terms of social work scholarship is not a linear process—we claim no clear starting point and continue to iterate through feelings of overwhelm, hope, and doubt. What sits at the core of this circularity are larger questions about the usefulness of ivory tower research as we know it, how to harness research in service of larger social justice goals, and about whether academic research is where we should be putting our efforts, resources, time and attention. *Isn’t there more pressing work to do?* One answer to this question is, honestly, yes. We are also in a window of opportunity for social work researchers to critically reflect and interrogate what it means for us to leverage this moment to push for a greater collective shift towards more timely, community-informed, and public scholarship. It also allows us to make space to consider: given the convergence of multiple global pandemics, what does it mean for social work scholars to be responsive in our research?

Prior to the current pandemic(s), there have always been social work scholars whose research has been deeply rooted in community needs, racial justice, and substantive interventions into social, economic, and health disparities. However, for many of us, our research questions and/or the context in which we ask them, should or may change as a result of this socio-political moment/movement. How should we engage with our previous research questions? How do we pivot our scholarship in a way that is meaningful and authentic? Should we be shifting our questions to the pandemic/protest context, and how the context exacerbates existing barriers/disparities? Or are there different, better, or more relevant questions such as *why* do we ask the questions that we ask? *What* and *who* informs our research questions? While often social work research emerges from community and practice-based needs, the current moment calls on us to more intentionally articulate how our research addresses the social injustices that have been exposed and magnified in the midst of these current pandemics. Social work is well-positioned to lead in this time, especially given our interdisciplinary knowledge base, our commitment to social justice, and our rootedness in applied and practice-based research. How can we leverage these strengths to promote Black liberation and community health and well-being on a global level?

## How do we do our scholarly work?

The conditions within which we do our work are changing as well, raising questions about *how* we engage in our research and scholarship. Over the past few months, with most of us sheltering-in-place due to the coronavirus pandemic, many aspects of our work have shifted. We see this in remote teaching and learning, Zoom meetings, children and family at home while we work, attending protests on our personal time, shifts in family employment, healthcare needs, and navigating the world in new ways to stay safe and healthy. It is undeniable that the rhythms of our work and lives are different now, and will be for the foreseeable future. As such, our current conditions also raise pragmatic questions about how social work research happens. How will our methods change? How do we collect data? What will we (and should we) ask of research participants? We may also be seeing shifts in our community partnerships as many agencies are experiencing unprecedented operational challenges in already difficult and under-resourced communities and institutional systems. Some community agencies are closing their doors as a result of the COVID-19 pandemic, others are stretched beyond capacity, and many are needing to scale back on activities that may be seen as “extra” such as research partnerships or hosting social work interns. How do we support community agencies in this time as researchers and educators committed to reciprocal and mutually beneficial relationships?

One of the things that has also been profound about these pandemics is the fact that people from all over the world are affected by systemic racism and COVID-19 in some way. None of us are outside of this context. Granted, the impacts are disproportionate and we are not all having the same experience, but it is still significant that we all are in this collective reality together. This calls into question some of the ways that we often position ourselves as researchers and educators—as somehow separate from students or research participants. Yet, in this moment/movement, our shared humanity is significant, regardless of our professional roles. Many of us are experiencing significant impacts of systemic racism and the COVID-19 pandemic and are also part of the communities that we do research with. How do we understand our roles in this moment/movement as researchers and community members? How do we also continue to engage with the demands of academic research and institutional expectations when our own lives and communities are directly impacted?

## Constraints and challenges

A thorough discussion of all of the macro and mezzo-level factors that make this kind of reflection and work challenging is beyond the scope of this essay. However, it is important to note we are raising these questions within the social/cultural context of these multiple pandemics as well as the context of higher education and, specifically, the market economies of research and social work—all spaces that have been shaped and constrained by white supremacy culture and neoliberalism. These forces significantly impact our ability to ask these difficult questions about the relevance and approaches to research as norms and expectations around productivity, external funding streams, and institutional expectations limit our ability to pause to reflect on what is truly needed right now in terms of our scholarship.

The current moment/movement we are living in provides an entry point to engage with these tensions, to think about them in new ways, and to potentially imagine and create new ways of doing our work. Despite our desire to ask these questions about our research in this political moment/movement, it is also important to be explicit that, none of the questions we have posed here are about fundamentally dismantling the (oppressive) structures of academia or research per se. However, it is our hope that our reflective engagement can help us to generate creative approaches for responding in ways that are useful, may serve as interventions into some of these broader macro forces, and can contribute to larger social justice goals.

## Where do we go from here?

In this essay, we are suggesting that the multiple crises we find ourselves in—namely of the COVID-19 pandemic (and its global health and economic impacts) and deep and enduring anti-Black racism—necessitate us as social work scholars to ask ourselves significant questions about our research. This moment/movement is providing an opening for us to make changes and to ensure that our work is responsive and useful to larger efforts for social, racial, and economic justice. We are seeing the need to grow our capacity to be with uncertainty and not knowing, to stretch around our learning and discomfort, and to challenge deep-seated systems and structures that we are a part of.

To close, we offer a few recommendations for further consideration by and for us as qualitative social work scholars:

- Create space and time for reflection and authentic examination of research processes, questions, epistemologies and methodologies in relationship to the COVID-19 health crisis, racial justice, and current sociopolitical contexts.
- Be thoughtful about how external funding sources impact our research interests and trajectories. Consider tensions between “chasing money” and being responsive to evolving social/political/cultural contexts, as well as tensions between academic career needs and community needs. How can we engage in shaping pandemic-related funding priorities and demand a justice lens in future research and funding agendas?
- Continue to interrogate our own positionalities, privileges, experiences of oppression, and how they shape our relationship to the current pandemics and all aspects of the research process.
- For white and other scholars from dominant culture groups: Read, learn, and integrate perspectives from Black scholars and other marginalized social work scholars that have long engaged in pandemic, crisis, and disaster-related work, who have been addressing structural anti-Black racism over time, and who have already been imagining community health and well-being in more holistic ways. Lift up (and cite) this work in ways that are meaningful but not extractive, co-opting, or performative in nature. Be aware of using citations by scholars of color as “virtue signaling” without it shifting anything about your work.
- For Black and other minoritized scholars: Practice self and community care, set boundaries, and find ways to navigate research when you may be balancing multiple roles, stressors, and impacts. Find and connect with like-minded scholars of color to create intentional scholarly community. Interrogate the terms of knowledge production within higher education and lift up (and cite) work by other minoritized scholars.
- Consider how and what we teach about research in all levels of social work curriculum. Discuss the current context as directly relevant to social work research. Examine what scholarship we use as foundational in other classes as well, such as those focused on social work history, policy, and human behavior—how are we reproducing dominant discourses rather than being responsive to current needs and challenges?

To ask ourselves these questions as social work scholars in this moment/movement has the potential to be scary, threatening, and challenging as well as invigorating, generative, and hopeful. In her piece on the pandemic, Ahmad (2020) writes:

Now more than ever, we must abandon the performative and embrace the authentic. Our essential mental shifts require humility and patience. Focus on real internal change. These human transformations will be honest, raw, ugly, hopeful, frustrated, beautiful, and divine. And they will be slower than keener academics are used to. Be slow. Let this distract you. Let it change how you think and how you see the world. Because the world is our work. And so, may this tragedy tear down all our faulty assumptions and give us the courage of bold new ideas.

It is our hope that this time of pandemics and protests, that asking real reflective questions about our research can help us to disrupt taken-for-granted assumptions about what we study and how, and can help us make our way into “the courage of bold new ideas”.
